# Cancer survivorship needs in Brazil: Patient and family perspective

**DOI:** 10.1371/journal.pone.0239811

**Published:** 2020-10-08

**Authors:** Antonio Tadeu Cheriff dos Santos, Rildo Pereira da Silva, Liz Maria de Almeida, Maria Lúcia Magalhães Bosi, Maria de Fátima Batalha de Menezes, Marcia Marília Vargas Fróes Skaba, Gustavo Nigenda, Carlos André Moura Arruda, Cleoneide Paulo Oliveira Pinheiro, María Cecilia González-Robledo, Felícia Marie Knaul

**Affiliations:** 1 Population Research Division, Nucleus of Research and Qualitative Studies, National Cancer Institute, Rio de Janeiro, Brazil; 2 Division of Population Research, National Cancer Institute, Rio de Janeiro, Brazil; 3 Laboratory of Qualitative Research and Evaluation in Health, Federal University of Ceara, Fortaleza, Ceará, Brazil; 4 National School of Nursing and Obstetrics, National Autonomous University of México, México City, México; 5 Centro de Investigación en Sistemas de Salud, Instituto Nacional de Salud Pública, Cuernavaca, Morelos, Mexico; 6 Department of Public Health Sciences, University of Miami Miller School of Medicine, Coral Gables, Florida, United States of America; 7 Institute for Advanced Study of the Americas, University of Miami, Coral Gables, Florida, United States of America; 8 Programa Universalidad y Competitividad en Salud, Fundación Mexicana para la Salud, Mexico City, Mexico; 9 Tómatelo a Pecho, Mexico City, Mexico; Institute of Mental Health, SINGAPORE

## Abstract

**Introduction:**

Cancer Survivorship is a growing public health challenge. Effective responses from health care and social services depend on appropriate identification of survivors and their families´ specific needs. There are few studies on survivorship in low and middle-income countries, therefore, more evidence-based studies are necessary to develop a comprehensive approach to cancer survivorship.

**Objectives:**

Identify the needs of cancer survivors and their relatives, specifically those of individuals with breast, cervical or prostate cancer, and with acute lymphocytic leukemia (ALL).

**Methods:**

A qualitative, exploratory study conducted in two referral institutions in Brazil, located in Rio de Janeiro (Southeast region) and Fortaleza (Northeast region). The study included 47 patients of public and private health services and 12 family members. We used script-based semi-structured interviews. The discursive material obtained was categorized and analyzed using the Thematic Analysis approach.

**Results:**

The analysis identified three central themes: 1) consequences of cancer treatment; 2) Changes in daily life associated with cancer survivorship; and 3) Unmet structural needs in cancer survivorship.

**Conclusion:**

Social and cancer control policies in Brazil should provide resources, specific care standards and clinical, psychological and social support. Cancer survivors should also receive rehabilitation and work reintegration guidelines. This matter requires broader access to qualified cancer information, development of an integrated patient-centered care and care model, and more research resources for the country's post-treatment cancer period.

## Introduction

Cancer survivorship is a complex phenomenon which requires specific and tailored approaches to planning, implementation and evaluation of care [1–3]. Cancer patients and family members experience a long-term major disruptive experience in clinical, emotional, and social terms; all of this requires unique norms for care giving, attention and protection of individual rights. A comprehensive identification of this population’s health needs is an essential initial step for planning whichever health actions are necessary to address the challenges as well as to increase the opportunities of cancer survivorship.

Although the concept of "cancer survival" is widely used today, the term is relatively recent and is attributed to Fitzhugh Mullan in 1985 [4]. Reflecting on his own cancer experience, he postulated the following relatively predictable survival stages:

(1) An acute phase from diagnosis until the end of the treatment. (2) Prolonged survival, beginning at the end of the first cancer treatment. It includes follow-up tests and consolidation or maintenance therapy. (3) Permanent survival, occurring years after cancer treatment, when recurrence may be less likely. [4] The value of these definitions and their extension to family members was great because it recognizes the enormous impact of a cancer diagnosis on patients and their families. This shock persists over time, regardless of medical treatment.

However, the definition of survival has remained a fuzzy concept today, with multiple meanings and debates about its use [5, 6]. Although the survival phase is recognized worldwide as part of the cancer continuum, it does not have standardized care protocols yet [7]. Over the past decade, efforts have been made to better define and develop categories for survival: Aziz emphasized the importance of incorporating late and long-term effects in the definition [8]. Miller et al sought to update Mullan phases in relation to treatments [9]. And Surbone who proposed four categories: acute, chronic, long-term, and cured [10]. The lack of a consensus on the survival concept and its phases has had consequences for patients, physicians, health systems and also for research, making it difficult to provide adequate clinical care and to develop public policies and research support [11].

In many high-income countries, the increase in cancer survival associated with advances in diagnosis, multi-modal treatment, and population aging has resulted in a growing number of patients living with chronic and late effects of the disease and/or its treatment, as well as with many associated social and economic consequences [12]. This has generated important reactions from research and service areas. It also draws attention and resources from the government, from non-governmental institutions, and from academics [13]. On the other hand, in many LMIC, access to health care–mainly for cancer diagnosis and treatment–has been the main focus of cancer control actions. These countries have been slow to recognize the specific needs of cancer survival, especially for those individuals in the post-treatment period. As a result, patients continue to experience significant barriers to accessing health facilities and to return to their daily life and work activities [14, 15]. Brazil fits this pattern. The Brazilian National Cancer Control and Prevention Policy considers five levels of intervention for cancer treatment: Prevention, Diagnosis, Treatment, Monitoring and Palliation [16]. However, there are no specific interventions to meet the needs of cancer survival.

We intentionally chose to study four types of cancer, seeking to focus on those most prevalent in the adult population, including users of both sexes. All of this, made the observation more comprehensive. As for ALL, its insertion brought the young population, composing the amplitude desired by the study. These types of cancer, therefore, allow us to assess the care needs offered to adults and children in Brazil today.

For 2020, 450,000 new cases of cancer are estimated in Brazil (excluding non-melanoma skin cancer). Breast cancer ranks first, with 66,280 new cases, followed by, prostate cancer with 65,840 new cases. Cervical cancer ranks sixth overall and third among women, with 16,590 new cases. Leukemias rank tenth, with 10,810 new cases [17]. The CONCORD III study estimated the five-year survival rate in Brazil, for the period of 2010–2014, at 92% for prostate cancer, 75% for breast cancer, 60% for cervical cancer, and 66% for Infant ALL [18]. The estimated number of prevalent cases (5-year), in 2018, in Brazil, for both sexes and all ages, were: 262,226 for breast cancer, 184,139 for prostate cancer, 46,858 for cervical cancer and 29,530 for ALL [19].

This scenario is similarly found in populations of other Latin American countries, especially considering the incidence, mortality and survival rates [18]. These numbers have direct implications for the people who have been diagnosed, for the societies and for health systems.

Brazil's Unified Health System (SUS) is one of the largest tax-financed public health systems in the world and offers free universal access to health services. SUS traditionally focuses on primary care, implemented through the Family Health Strategy (FHS). Through the FHS, SUS provides integrated primary care to two-thirds of the approximately 206 million Brazilians [66]. A quarter of Brazilians have private health insurance [20], resulting in health expenditures of US$ 105 billion in 2014. These expenses, by comparison, exceeded total government spending on health at $ 90 billion [20]. The Brazilian health system, which is likely the best in Latin America, is theoretically capable of offering the necessary services in the survival phase [16, 20]. However, in practice, there are challenges to offering this type of care [20]. The main challenge is that survival is still not recognized as an important issue in Brazil. The system was designed to refer patients seen in the primary care units to more complex secondary and tertiary services. But this is not happening, which is partly due to the economic slowdown as well as to lack of training and experience of primary care teams in managing patients with a cancer history [20].

There are few studies on cancer survival conducted in LMICs, including Brazil, using a qualitative approach. In 2018 the IARC [19], estimated a 5-year prevalence of 1,307,120 cases in Brazil. The overall objective of this study was to identify cancer survival needs, drawn from the perspective of cancer patients and their families. Specifically breast, cervical, prostate cancer and acute llymphocitic leukemia (ALL).

## Method

The study applied a qualitative approach, within the interpretive tradition, considering the subjective experiences of individuals who had a cancer diagnosis and their families [21–24]. This approach was adopted to explore and understand the meaning that was given to the survivorship phenomenon [25].

The study was conducted in Rio de Janeiro (from May to August, 2014) and Fortaleza (from January to March, 2015), located in the Southeast and Northeast regions of the country, respectively. These regions differ in geographic, socioeconomic and cultural aspects as well as in the level of healthcare network organization. The cities are two large Brazilian capitals that nationally rank second and fifth in terms of number of inhabitants, with population estimates in 2017 of 6.5 and 2.6 million inhabitants, respectively.

The fieldwork was done in public and private health institutions that offer oncological care: “José Alencar Gomes da Silva National Cancer Institute (INCA)” (public) and “Integrated Oncology Clinics (COI)” (private), in Rio de Janeiro, Walter Cantídio University Hospital (HUWC) (public) and “Ceara Cancer Institute (ICC)” (private), in Fortaleza.

Participant selection was done through purposive sampling. The interviewers reviewed medical charts and chose those participants who, after a previous telephone contact, agreed to participate. Interviews were conducted when patients returned to health facilities for follow-up appointments, (they had been diagnosed at least one year before). Interviews, both of family and patients, were conducted individually in private rooms in the hospital, with an average duration of 40 minutes. We reached forty-eight survivors who had been diagnosed at least one year before the time of the interviews; twelve for each cancer, (six in the public health system and six in the private health system). We also contacted 12 family members (three for each type of cancer in the public and private sectors but not necessarily family or blood-relatives. Those who chose to participate in the study signed an Informed Consent Form.

The inclusion criteria considered: (a) patients diagnosed with breast, cervical, prostate cancer and acute lymphocytic leukemia, aged 18 years or older, who had been diagnosed at least one year before the time of the interviews. (b) Spouses or significant caregivers of cancer survivors, aged 18 years or older. The criteria of ¨at least one year since diagnosis¨ considered those who had accumulated sufficient experience with the disease, to be able to discourse on the subjects of interest. All individuals in end-of-life care and those with cognitive difficulties to participate in the interview were excluded.

The discursive material was obtained through semi-structured individual interviews, using a semi-structured script that was specifically designed for the study. It was inspired in Mullan,’s stages of survivorship [4], containing diagnosis and treatment; significant moments in diagnosis and treatment; changes in daily life; type of information and guidance received during treatment and after treatment; type of emotional and social support received, and suggestions for the health system, based on the participant's experience.

The structuring of the interview guide was done after a pre-test with cancer survivors and their families. Open-ended questions were designed to access illness narratives according to existing literature [26–28]. The interview guide underwent forward and back-translation and was adapted to the Brazilian reality (ATCDS, RPS, MFBM, MMVFS, GN). Interviews were conducted by five health and social science experts who had wide experience in qualitative research fieldwork in both social and oncological fields (MFBM, MMVFS, MLMB, CAMA, CLPOP). Procedures allowed researchers to obtain primary data from personal testimonies, and to stimulate free verbalization about the inherent needs of cancer survivorship. Given the recent criticism of the theoretical saturation principle and because we defined the number of interviews prior to the study, the sample closure was based on the principle of "information power" [29], which is more congruent with the tradition to which our research is affiliated.

The initial categorization of the discursive material was performed in each city by the same researchers who conducted the interviews. The interviews were transcribed literally. The authors, working independently, validated each transcript as a rigor criterion. The categorization was built, based on recurrences of the statements enunciated in the transcriptions and convergences and divergences of their meanings. We based our analysis on the thematic discourse approach, which is aligned with a critical interpretative approach, in order to articulate the socio-cultural and clinical contexts and enable the interpretation of qualitative data [30–35].

The categorized and interpreted material was subsequently re-analyzed by all authors in a workshop in Rio de Janeiro in 2018, in order to integrate and re-evaluate the material gathered in different cities, as well as to validate the interpretation together. The themes were equally defined and refined for the final version of the results and discussion in the article.

The speech fragments of individuals diagnosed with cancer and their family members were coded as follows:

- Group of respondents: P (patient) and F (family)
- Types of cancer: C (cervical), M (breast cancer), L (leukemia), and P (prostate cancer)
- Type of health unit: A (public); B (private)
- Interview number: 1–24
- Research site: R (Rio de Janeiro city) and F (Fortaleza city).

This study was approved by the Research Ethics Committees of the National Cancer Institute (dossiers: 523.004; 555.869; 560.305; 801.666) and by the other participating institutions (Research Ethics Committee of Hospital Pró-Cardíaco by the “Integrated Oncology Clinics (COI)”, and Research Ethics Committees of Walter Cantídio University Hospital (HUWC) and the “Ceará Cancer Institute (ICC)” [36].

## Results

The study population consisted of 59 individuals: 47 of whom were diagnosed with cancer (prostate (12), breast (12), cervical (12), and ALL (11)), and 12 family members (three for each type of cancer), who were in, or accompanying treatment in the health institutions that offer oncological care in Rio de Janeiro and Fortaleza.

Among those who were diagnosed with cancer, 64% were female and married, and about half of them were 60 years old or older. 43% had a higher education level and over 85% had income from paid activity. Approximately 36% were treated in the public health network, 45% were treated in the private network, while 19% used both networks. Approximately 36% and 45% were treated only in public health and private networks, respectively, and 19% in both networks.

The median time between diagnosis and interview for individuals diagnosed with cancer was 3.1 years, with a minimum of 1 year and a maximum of 18 years. In Rio de Janeiro and Fortaleza, the median time was 3.1 years (1.5–18 years) and 2.5 years (1–14.6 years), respectively. For 37% of individuals with cancer in Rio de Janeiro and 21% in Fortaleza, the interval between diagnosis and interview was >5 years. In Rio de Janeiro there were 2 individuals with breast cancer (66 and 70 years old); 3 with prostate cancer (56, 73, and 74 years old); 2 with cervical cancer (66 and 70 years old) and 2 with ALL (both female, 21 and 23 years old). In Fortaleza, there were 3 with breast cancer (61, 64, and 64 years old); 1 with cervical cancer (74 years old) and 1 with ALL (female, 35 years old). The remaining patients had been diagnosed between 1 and 5 years before the interview and had already completed primary treatment.

Family members interviewed in Rio de Janeiro were between 31 and 71 years old and those from Fortaleza were between 30 and 85 years old. Interviewees who were not related to patients from our study, had relatives with the following types of cancer: Rio de Janeiro (02 with cervical cancer—38 and 70 years old; 02 with breast cancer—31 and 52 years old, 01 with ALL—35 years old, and 01 with prostate cancer—71 years old). Fortaleza (02 with cervical cancer—45 and 85 years old; 02 with prostate cancer—56 and 79 years old; and, 02 with ALL—30 and 33 years old). More than half of the family members were spouses (58%); 67% were female, 58% were over 40 years old, and half of them had completed elementary school. More than 65% of the respondents had a paid activity. The median time between the relative's diagnosis and the family's interview was 3 years, with a minimum of 1 year and a maximum of 14 years. In the city of Rio de Janeiro, the median time was 08 years (02–14 years) and, in the city of Fortaleza, it was 2.5 years (1–6 years). Their relatives had already completed primary treatment.

### Interpretation structure

The analysis focused on three central themes: 1) Consequences of cancer treatment; 2) Changes in daily life associated with cancer survivorship; and 3) Unmet structural needs in cancer survivorship.

#### 1. Consequences of cancer treatment

The responses that participants gave articulate objective dimensions as well as psychological and symbolic aspects. These are reports that indicate physical, psychological (emotional), and socioeconomic needs arising from living with and beyond cancer diagnosis and treatment. This set of aspects shows how the individual and his family and community are affected by the consequences of the disease.

Physical issues are mainly related to the repercussions of treatment, such as complaints of pain, fatigue, constipation, urinary problems, and other specific complications of radiotherapy, chemotherapy as well as other treatment aspects, as described below.

I had a lot [fatigue] from chemo [therapy] and radiotherapy. (PMB10R)

I spend the day wearing a diaper because, otherwise, underwear would not be enough. The dripping (of urine), which persists, causes certain discomfort. (PPA21F)

The results showed that the most common problems that participants mentioned were the physical and emotional needs, related to the repercussions of the disease and its treatment. This constitutes one of the main challenges that health care networks face when addressing cancer survivorship.

As for the emotional dimension, individuals with a cancer diagnosis referred to the awareness of the transience of life and, at the same time, the joy of being alive. Despite the fear and the intercurrences, apprehensions about the possibilities of cure or death functioned as a unifying element among the family and their affective bonds, as stated below.

I have seen the transience of life. I have seen that you can be very well, in full activity and suddenly something comes up that knocks you down. I do not want to give importance to certain things anymore. I will live for my family; I will live for my grandchildren. (PCB06R)

[…] the disease taught me to value my life more. […] And the worst [situation] was when I thought about my kids. (PMB12R)

On the other hand, the families reported an enormous burden associated with caring for the individual with a cancer diagnosis, as described in the narratives below:

Today, I am always by her side… I leave everything behind. I leave my work, to be able to be with her. She loves it, she says to me: 'It is very good to be with you'. The love between us is stronger. We have become much closer. (FMA03R)

I do not have a mother; I do not have a sister. There are people who can look after him, but I think in his situation [mother of a child with ALL] he needs someone more present. [FLA05R]

For me, it is good to have this conversation we are having. I also think that not only for the patient, but also for the relative of the patient who is the closest, because it ends up causing a lot of harm to me. (FCA01R)

Family members and individuals diagnosed with cancer reported socioeconomic difficulties related to treatment follow-up, such as the completion of complementary exams, purchase of support medications, and resources for transportation and food. The following testimonies describe this point:

So, [there’s] the expense of, the medication she must take home. […] there is transportation, right? (FLB06F)

My mother borrowed money to pay the person who came with me and also buy the food. (PCA02R)

[…] he also sold an object that he had. He sold it and paid [for medicines and tests]. (PMA07F)

#### 2. Changes in daily life associated with cancer survivorship

Individuals who received a cancer diagnosis emphasized the importance of a diet, physical activity and leisure to stay healthy. Some family members reiterated that the diagnosis and treatment of cancer motivated them to change their own health care behaviors. More specifically, both groups mentioned adherence to healthier habits and the need for regular routine exams for prevention and early detection of cancer. Examples are as follows.

[…] I am taking better care of myself. I began doing not only the mammogram; in general, we started to rethink that too. (FMB04R)

Now she takes better care of herself […] I believe that after she leaves here she will continue to comply with them [the tests], (FCA01R)

The changes underscored by the informants express a very relevant meaning in the survivorship process. The experience of illness imposed changes in several areas of daily life for those with cancer and their families. The narratives indicated the occurrence of changes in the relationships and social roles played by fathers, mothers, and children, and restrictions on leisure habits, as described below.

I can no longer carry heavy loads. I am not in shape to do so. I cannot dust the house. I cannot do the laundry. I can't do any of that, as it makes me move my arm too much. (PMA09F)

Today I'd say the roles have reversed. Now I am like her mother and she is my daughter. […] She became very dependent. She doesn’t stay at home alone. She’s afraid of feeling unwell […]. (FLA01F)

I’ve always liked dancing, she’s always liked dancing, and we stopped dancing because of that. She cannot dance. She has a hard time walking now. (FCB02R)

The narratives of individuals who have been diagnosed with prostate and cervical cancer and their families highlighted the late effects of treatment, especially restrictions on their sex life, as follows.

I need sex. How do I do this? They [the doctors] started trying different treatments, but none of them met my needs because they all caused damage. They said: “Look, your solution will be a penile implant”. I [said], “Ok, whatever you want to do, as long as you fix my problem”. Because without an erection what sexual act can be practiced? None. (PPA21R)

After removing the organ, it is not even possible for you to do a transvaginal [ultrasound scan]. Because [any penetration] causes bleeding, there’s no way. Then there was a talk with the doctor. She said: “Look, you need to look for other resources [for your sex life]”. (PCB04R)

Yes, I think the one who needed to do psychological treatment was me (…). She told me she had lost her sexual appetite; she has no more [sexual] desire. Sometimes she does it to make me happy. Sometimes it’s not good, and it makes things confusing between us… From this treatment, without a doubt, our biggest problem. If the husband does not have love, affection and patience with his wife, I think the marriage starts being in trouble. (FCA01R).

For family members, changes resulted in the need to reorganize family routines and work schedules. In one case, it was necessary for the family member to resign from work, as in the case of the mother of a patient with ALL:

[…] it was complicated because she spent more than a month hospitalized, so we had to miss work, he is dependent. […] it was a double burden… I had to leave my job, and I probably cannot go back so soon… it was very hard to make that decision. "(FLB06F).

#### 3. Unmet structural needs in cancer survivorship

Family members and individuals diagnosed with cancer who used public and private healthcare, stressed the need for specialized care, focused on the patient's needs. They mentioned aspects, such as improvement in the structure of access to health units, economic support in transportation, food and pharmaceutical expenses during treatment, and post-treatment social security advice. Consider the following statements:

Even after treatment, I think you should still have support from your family and doctor. She will always need this support. They will always need to be [re]evaluated. (FCA01F)

I suggest differentiated care at the medical level […]. Financially for us. This is fundamental. (FCB02R)

[…] sometimes [they think]: 'Well, the guy is in private care, so he can afford.' No, sometimes he is in private care because of his work. Normally, I would not be able to afford a (Private) Health Insurance for the four of us [the wife and the two children]. So, [they think] if the person has a (private) health insurance, why would we help with the medication? (PLB16R)

It is very difficult to transport people. So, [you need] more financial support because of the treatment, because of the food, because it gets in the way of your finances. Better government social support [like] food stamps. And, depending on the location of the residence, transportation to take to and from. (PCB01R)

… It is the issue of the location, to have access [to the health service] over the weekend, because, as I told you, […] many times, what upsets you when you arrive at a Health Care Service is that you need to be taken care of, but you don’t receive the quality care you expect. "(FLA05F)

Another need that was informed refers to the possibility of receiving continuing care for mental health issues associated with the emotional effects of cancer survival, as shown in the following statements.

Public power [government] must provide support, because the emotional [aspect] is very shaken. (PCB06R)

[…] I think there are a lot of people [who need support], [because] they still have this fear, the fear that the disease will come back. (PLA13R)

These individuals also pointed to the need for guidelines and information on the consequences of the disease in its clinical aspects (control of symptoms and questions about sex life), social aspects, and economic aspects (social security rights and return to work).

No, this [the post-treatment guidelines on sex life] has not been passed on to us, even yet… It was just mentioned […] the doctor said that she has to loosen up more. […]. (FCA01R)

They did not give me much [information], just said that I would have a fever and would vomit (PLA14F).

I received some guidance, but after the treatment, you adapt according to the orientation you were given, you are living this [new] moment (PMA07R).

I do not know what is like to protect the worker who has cancer at work. I never asked anyone about the legislation. If I leave my job, I will not be able to get work elsewhere. I do not have medical discharge. So I think the company cannot fire me, I do not know how it works "(PLB16R).

Specifically for the public network, participants report the need for improvements in the process of having access to diagnosis and treatment, the quality of complementary examinations and the structure and availability of vacancies. They exemplify these demands for integrated and quality care with a proposal to expand the cancer care organization, by creating diagnostic support units that have INCA’s recognition of quality standards:

They have to change everything. In the preventive area. The time from the medical appointment to the exams. The referral to the specialist is very long, […]. The quality of the complementary exams is sometimes good but not always. (FMB04R)

[…] Sometimes the person cannot survive because of this waiting period. There are people who underwent surgery and had to wait three to four months to start radiotherapy, and time, when you suffer from this disease, I think is very important. (PMA07R)

[…] I think there is still a very large shortage of hospital beds, you know. You realize that if people are in that sector of hematology and if they are there, it’s because of health problems (…) the [human] contact can kill, there are three people in the same room, three hospital beds in the same space where even the ventilation is complicated because the three patients and companions remain there (…) (FLA05F)

First, there would have to be a reference center. […] many things could be changed in the INCA, there could be a pre-INCA (Cancer diagnostic center), or […] construct other INCAs, because you know that INCA, as a treatment center, is even better than some private ones. (PLB16R)

Regarding private health units, demands were related to the improvement and agility of the authorization process of health insurance operators for treatment procedures. Each procedure requested by the doctor, required authorization from the operators, which led to delays in access to medical procedures, tests, medications and surgeries. Thus, comprehensive care, centered on the patient´s needs, is often not achieved because of bureaucracy, as exemplified by the following statements:

For us (private health care users) who have been assisted by a private health insurance, sometimes something more urgent depends on an authorization from an auditor, it is more complicated […]. (FLB06F)

I needed to be hospitalized, it was fast. Because if our government, our health system had an evaluation of each case, the treatment, which is very painful, could be improved. (PLB16R)

## Discussion

The discussion of the study is developed around three central themes defined for the interpretation of the results: 1) Consequences of cancer treatment; 2) Changes in daily life associated with cancer survivorship; and 3) Unmet structural needs in cancer survivorship. In all these topics, the interviewees indicate that the cancer experience is a long journey, not only in search of a cure and / or treatment, but also in a search for mitigation and support for the clinical, emotional and social impacts resulting from the consequences of cancer treatment [4, 37, 38]. Cancer treatments, as reported in the literature, may cause physical, emotional and / or socioeconomic changes either immediately or later in life [39–44]. These aspects were highlighted in the reports of the study participants. Although they have different socioeconomic and cultural characteristics, residing in different cities and attending by non-congeneric public and private health care, reported similar reactions regarding, they reported similar complaints and problems. It is relevant to highlight that the group interviewed, although it had participants with more than five years of post-treatment, was composed mostly of individuals who were still under the impact of the consequences of primary cancer treatment and in transition to the follow-up period.

### Consequences of cancer treatment

In the study, the first theme refers to the immediate and long-term consequences of cancer treatment. There are complaints of pain and fatigue, limitations to activities of daily living and changes in sleep, nutrition, excretion and sexual functions (especially in cases of prostate and cervical cancer). It is important to be highlighted is that some of these spheres of life (notably sexuality) are not systematically included in cancer treatment protocols. As a result, social withdrawal, sexual life limitations and disruptions in daily life, including returning to school or work, are identified as pressing needs for attention and care.

These consequences produce important emotional impacts that also require a specific level of care [45] that must start from the diagnosis and extend through all phases of treatment and post-treatment [46]. Once primary treatment is completed, follow-up is a process that can be long, depending on the type of cancer. Clinical visits to the oncologist often produce feelings of helplessness and distress, as they result in a multitude of worries and fears [41]. These fears and concerns result from the risk of relapse, the occurrence of other cancers, physical sequelae, late toxicity caused by treatment, physical deterioration and death. These fears and concerns in surviving patients constitute the “Damocles or Lazarus Syndrome”, a state of liminality that can extend throughout life, referring “not only to psychological suffering due to vulnerability to the disease, but also to the difficulty of develop or have a long-term life project” [47].

The healing and adaptation processes of these individuals involve all scars—physical and emotional—during the phases of diagnosis, treatment, and post-treatment and reflect a “biographical rupture” [48–51]. When seeking professional help, many patients hope to understand how their daily lives have changed [49]. That is, “the patients' attempt is to seek strategies for reorganizing practical life that give a new meaning to their life” [52, 53]. On the other hand, caregivers can go through the same process, to a greater or lesser extent.

Thus, attention to the emotional dimension, as suggested by the participants, is extremely important, because it comprises (together with specialized medical care) the basis for adaptation to the post-treatment phase [46]. Evidence from the European Guide to Improving Quality and Comprehensive Cancer Control (Cancon) [40] indicates that early psychological interventions contribute to the reduction of psychological morbidity and improve disease adaptation, quality of life and well-being post-treatment. Although several countries such as England, France and the United States have developed clinical guidelines for use of evidence-based psychosocial care, this practice is not yet routinely employed in Brazil’s health care programs.

With regard to the economic dimension, the labor issue and the return to work activities after treatment are highlighted. Resumption of work activities is considered an important aspect for maintaining and improving the quality of life [40, 41]. Health care providers, employers and society-at-large all have a role to play in facilitating reintegration into work. In Brazil, labor legislation does not recognize the specific needs of individuals diagnosed or disabled due to cancer in the workplace work environments [54]. For example, labor law does not provide working mothers a paid leave to accompany their sick children. This contradicts the Statute for Children and Adolescents (Law No. 8,069, of July 13, 1990), which determines that those responsible, usually their mothers, have the right to accompany hospitalized children [55]. The decrease in working hours or loss of employment and the consequent reduction or loss of family income increases the vulnerability of cancer survivors and their families [56, 57].

Thus, the emotional and socioeconomic needs highlighted by the interviewees strongly recommend improving the organization of the health care network and social assistance services. Concerning to these issues, it is recommended to providing information on social and social security rights and developing specific social and health policies [38, 58] as well conduct intersectoral actions and to mobilize organized civil society.

### Changes in daily life associated with cancer survivorship

The second theme that emerged from our analysis pertains to daily changes related to cancer survivorship. Participants indicated a willingness to adopt healthier living habits, periodic preventive examinations, and greater dedication to family and friends. Participants and family members also reported the need for psychosocial support, a demand that often goes unnoticed by the health care system and by social policies in Brazil. Needs reported by the participants refer to the right to have access to clinical and psychological treatment and coverage of medication costs, transportation, and nourishment of individuals undergoing treatment/follow-up care and their families.

Faced with a worldwide scenario in which the number of cancer survivors is growing steadily, this assessment, from the perspective of patients, in terms of health-related quality of life, are essential indicators for the organization and planning of policies and health care in Brazil. As in developed countries [59, 60], the most significant increase in survival occurs among the elderly. In 30 years, Brazil is expected to reach a population of around one million people per year in the age group ≥65 years, forming an age pyramid similar to that of a developed European country [61]. This trend has the consequence of the need to prepare Brazil's health systems for the survival phase [15, 38] with an emphasis on services and care lines that effectively improve health-related quality.

Psychosocial support will require a person-centered approach, based on each individual’s situation: diagnosis and prognosis, medical and non-medical treatment, intrapersonal and interpersonal factors, patient’s values, aspirations and priorities and peer attitudes. To understand the experience of chronic diseases, health care providers must realize that managing post-treatment cancer control is not only a matter of planning appropriate treatment prescriptions, but also a process that integrates and understands social interactions and daily activities [48, 49]

### Unmet structural needs in cancer survivorship

The third theme covers unmet needs related to access to the public and private health care system, improvement of infrastructure, and processes organized by attention levels [62–64]. In the public sector, suggestions pointed to coverage and access to timely treatment, while in the private sector they were related to the control that health insurance companies have over authorizations of procedures recommended by specialists [62, 65].

In the private sector, there is an offer capacity, although in practice the issue of payment methods and authorizations of services and treatments does not favor quick and immediate access to the necessary care. Its users, mostly middle-class employees, face restrictive and time-consuming health insurance authorization processes that often cause legal demands [20].

Considering the epidemiological profile of cancer in Brazil, these demands require urgent restructuring and planning, especially for the organization and use of survival care plans.

According to the US National Cancer Institute, a survivorship care plan is “a detailed plan for follow-up of patients after the end of their treatment” [66, 67]. A survivorship care plan may include a schedule for medical clinical visits; monitoring of health problems that may arise after the end of treatment, and offering information to meet patients’ emotional, social, legal and financial needs. It should also include referrals to specialists and recommendations for a healthy lifestyle.

United States and Western Europe employ two main models to organize their care systems: the Shared Care model, integrating specialized care and the core network and the Specialized Survivorship Clinics [40, 41, 67].

The choice of the model depends primarily on the structure and resources of the health system, including the role and qualification of primary care agents and the reimbursement/insurance coverage scheme [68]. Although the evidence shows their value to patients, providers, and health care systems, cancer survivorship care plans are still underutilized and very few cancer patients have access to them, especially in low and middle-income countries. In Brazil, the challenge of developing and implementing a cancer survivorship plan must be a health care priority. The first action should be using data from population-based cancer registries [20], which would provide information about number of survivors and their possible needs for post-treatment care. The second action would be planning and implementing comprehensive care, with multi-professional teams who are focused on care of physical, emotional and social consequences of the disease and its treatment. The investment in training these teams will probably increase quality and cost-benefit of care in the survival phase. [20].

Concerning actions targeting socio-economic aspects (economic, social welfare and reintegration and rehabilitation at work), it is recommended to conduct intersectoral actions and to mobilize organized civil society, given the world-wide increasing rates of cancer incidence and prevalence [19].

## Conclusion

Considering the themes and meanings highlighted in this study, it is urgent to plan and implement lines of comprehensive care in the cancer post-treatment in Brazil. This model must be suitable to address the mid and long term, physical, social, and economic consequences of cancer and its treatment.

Comprehensive care should emphasize affective and symbolic aspects of survivorship as essential for the organization of humanized care models, focused on the specific needs of individuals affected by the disease and their relatives. Issues related to cancer survivorship must be included in current cancer policies as well as national agendas for health and social well-being.

### Study limitations

This study was exploratory and descriptive in nature. The results indicate the need to conduct additional studies covering other types of cancer and individuals who were diagnosed and treated more than five and ten years before interviews.

## Supporting information

S1 File (DOCX)

S2 File (DOCX)
