# Supplementary material for: Cancer survivorship needs in Brazil: Patient and family perspective
Source: PLoS One. 2020 Oct 8;15(10):e0239811. doi: 10.1371/journal.pone.0239811 (PMC7544129; doi:10.1371/journal.pone.0239811)
Supplement: S1 File — (DOCX) [file pone.0239811.s001.docx]

Individual interview guide for family survivors

Breast cancer, Cervical Cancer, prostate and acute lymphoblastic leukemia

(Age or older than 16 years)

Semi-structured script

Name of Interviewer: _________________________________________________________ _

Interview Location:State ____________ Municipal______________ Unit:_____________

Date and time of Interviw: ____/____/____; ________________

THANK YOU FOR ACCEPTING THIS INTERVIEW. QUESTIONS THAT WILL BE FORMED TO YOU ARE DIVIDED IN SEVEN SECTIONS: 1) GENERAL INFORMATION, 2) DIAGNOSIS, 3) TREATMENT, 4) SHORT AND LONG TERM EXPERIENCE WITH CANCER, 6) SUPPORTS RECEIVED, 7) EXPENSES WITH DISEASE AND 8) BALANCE SHEET OF EXPERIENCE.

1. General Information (contextualization)

1.1. Name of interviwee: (code)____________________________________________________

1.2. birth: Date ____/____/____ State______________ Municipal___________________

1.3. Sex: ( ) male ( ) female

1.4. Marital status:

( ) married ( ) divorced ( ) separeted ( ) judicially separately ( ) widoer ( ) single ( ) stable union

1.5. Scholling (high level course):

( ) Elmentary school ( ) High school ( ) College ( ) Postgraduate studies

1.6. Do you Have work paid? ( ) Yes ( ) No

1.7. What kind of occupation do you have? _____________________

1.8. it is user of :

( ) Public health service ( ) Private health service ( ) Both

1.9. What is your relationship with the patient: _______________________________________

1.10. Family structure (how many people live with the same income ?):

( ) 1 ( ) 2 ( ) 3 ( ) 4 ( ) More than 4

1.11. Do you live in the same residence that the patient? ( ) Yes ( ) No. Case no, number of comforts in your home (beyond the kitchen and bathrooms: ( ) 1 ( ) 2 ( ) 3 ( ) 4 ( ) More than 4

1.12. Residence location :State___________________ Municipal___________________

1.13. History of cancer in the family: are there cases of cancer in your family? ( ) Yes ( ) No

1.14. History of diseases: Was he a healthy person before the diagnosis? ( ) Yes ( ) No

1.15. Does/Did he have constant health problems? ( ) Yes ( ) No

1.16. Did he extended any treatment? ( ) Yes ( ) No

1.17. When did your family receive his/her cancer diagnosis (date)?____________________________

1.18. ~~I~~which type of health unit did he receive the diagnosis?

( ) Basic public health unit ( ) Public hospital

( ) Private doctor´s office ( ) Private hospital

1.19. Location of health unit: State __________________ Municipal_______________

1.20. How long was between the diagnosis and treatment beginning? ____________________

1.21. Date of initiation of treatment (first therapeutic intervention): ____/____/____

1.22. How long was treatment/how much time does treatment?______________________

1.23. Does he continu to treatment? ( ) Yes ( ) No

1.24. What is his treatment?

( ) Chemotherapy ( ) Radiotherapy ( ) Surgery ( ) Hormiotherapy ( ) bone marrow transplantation

1.25. How often did he go to health unit at the treatment phase?______________

1.26. Did it brought complications for him? ( ) Yes ( ) No. Type:­­­­­­­­­­­­___________________________

1.27. What kind of support was offered to your familar during his visit to the health unit?

| unity health | ( ) Physical | ( ) Emotional | ( ) social |
| --- | --- | --- | --- |
| family | ( ) Physical | ( ) Emotional | ( ) social |
| civel society | ( ) Physical | ( ) Emotional | ( ) social |
| social protection | ( ) Physical | ( ) Emotional | ( ) social |

1.28. Have you been informing about the diagnosis and cancer treatment that your relative have been receiving?

( ) Yes ( ) No

2. Diagnosis of illness (audiography)

2.1. Could you describe how your family was feeling after the diagnosis (description of physical and emotional feelings, and possible changes in time)? Did he received any kind of physical, emotional,and financial support,? (how do you evaluate the weight of each one)? Could you describe how you feel after the diagnosis (description of physical and emotional feelings, and possible changes in time)? Did you receive any kind of, physical, emotional and financial support? (how do you evaluate the weight of each)?

3 .Treatment (audiography)

3.1 Do you consider that the treatment of your relative was satisfactory? _________________Why?. (explain the reasons why you think that the treatment was satisfactory or unsatisfactory)?

3.2. Could you tell us about the consequences (positive and negative) that the treatment had in the disease evolution of your relative in their respective stages?

4. Short and long term experience with cancer (audiography)

4.1. Could you appoint what were the most important (significant) moments for you and your relative during the cancer experience?

4.2. What are the moments that you most remember (explore the meanings)?

4.3. Has your relative stopped smoking or intake alcoholic drink? Has your relative changed his food habits?

5. Activities before and after treatment (audiography)

5.1. Did you receive any information in the health unit about the changes that your relative would have after (or also during) the treatment? Did you receive any orientation how to deal with these changes?

5.2. Can you describe the positive and negative changes (physical and emotional) observed in the life of your relative since the cancer diagnosis? Can you tell for us how these changes affect the everyday life of your relative and your family?

5.3. Does your relative do any activity that he did not before his cancer diagnosis?

5.4. Can you describe for me one day of the week and one normal sunday before and after the cancer diagnosis of your relative? What has changed?

5.5 did your relative return to do all the activities that he did before treatment (individual, family, and social)?

5.6. If your relative returned to work and / or to go to school, please talk about the possible adaptations that was necessary for that happening (whole or partial time, with mobility and accessibility mechanisms, etc).

5.7. Can you talk about the reaction of friends (in the work and/or school) with your relative?(support, solidarity, etc indifference)?

5.8. did you need do any adaptation in your home since the treatment for a better confort of your relative (furniture, bathroom, kitchen etc)?

5.9. Did you need to redistribute any home tasks between family members (cleaning, shopping, taking children to school etc)?

6. Supports received (audiography)

6.1.Between diagnosis and treatment, what kind of support has your relative received (of physicians, health units, your family or non-governmental organizations?

6.2. Who in your family carry out especially from patient care? How?

6.3. Other family members have participated in patient care since the diagnosis?

6.4. How was your relationship with your relative after the disease?

6.5. How was the relationship of the other members of your family with the patient?

6.6. How do you think he feels now? How do you think he has been feeling late (weak or strong, sad or glad)?

6.7. Did they prescribe to him medications in the health unit? What did they prescribe? Does he take another type of medicines?

6.8. Does he receive drugs to feel better? Other treatments? Do you think that he needed or need more help from physicians, health units, your family or non-governmental organizations to get better his life today? What kind of help?

6.9. If you could ask for any things to help your relative to live better since the diagnosis, what would them be (beyond direct medical attention)?

7. Expenses with disease (audiography)

7.1. From the diagnosis, have you need to pay for medicines, laboratory, professional support or others?

7.2. How had your relative done to cover the expenses (betake to savings, loans etc)?

7.3. Does the expenses with disease have affected the economic situation of your family (describe)?

8 . Experience balance: (audiography)

8.1.Would you know to tell me why your relative had this disease?

8.2. Do you consider that you had any gain (wide amount)?

8.3. Would you like to suggest or make any recommendation for the people who treated your relative and head units and health policies?

8.4. What would you like to tell for a person who receives today the same diagnosis of the illness that your relative had?

8.5. How do you see the life of your relative currently?

8.6. How do you see the life of your relative in the future?
