# Supplementary material for: Cancer survivorship needs in Brazil: Patient and family perspective
Source: PLoS One. 2020 Oct 8;15(10):e0239811. doi: 10.1371/journal.pone.0239811 (PMC7544129; doi:10.1371/journal.pone.0239811)
Supplement: S2 File — (DOCX) [file pone.0239811.s002.docx]

GUIDE OF INDIVIDUAL INTERVIEW FOR SURVIVORS
BREAST CANCER, CERVICAL CANCER AND PROSTATE AND ACUTE LYMPHOBLASTIC LEUKEMIA

(AGE OR OLDER THAN 16 YEARS)

SEMI-STRUCTURED SCRIPT

Name of Interviewer: _________________________________________________________ _

Interview Location:State ____________ Municipal______________ Unit:_____________

Date and time of Interviw: ____/____/____; ________________

THANK YOU FOR ACCEPTING THIS INTERVIEW. QUESTIONS THAT WILL BE FORMED TO YOU ARE DIVIDED IN SEVEN SECTIONS: 1) GENERAL INFORMATION, 2) DIAGNOSIS, 3) TREATMENT, 4) SHORT AND LONG TERM EXPERIENCE WITH CANCER, 6) SUPPORTS RECEIVED, 7) EXPENSES WITH DISEASE AND 8) BALANCE SHEET OF EXPERIENCE.

1. General Information (contextualization)

1.1. Name of interviwee: (code)____________________________________________________

1.2. birth: Date ____/____/____ State______________ Municipal___________________

1.3. Sex: ( ) male ( ) female

1.4. Marital status:

( ) married ( ) divorced ( ) separeted ( ) judicially separately ( ) widoer ( ) single ( ) stable union

1.5. Scholling (high level course):

( ) Elmentary school ( ) High school ( ) College ( ) Postgraduate studies

1.6. Do you have work paid? ( ) Yes ( ) No

1.7. What kind of occupation do you have? _____________________

1.8. it is user of :

( ) Public health service ( ) Private health service ( ) Both

1.9. Member’s families list, kinship and age:

| Famlies | Kindship | Age |
| --- | --- | --- |
|  |  |  |
|  |  |  |
|  |  |  |
|  |  |  |

1.10. Family structure (how many people live with the same income?):

( ) 1 ( ) 2 ( ) 3 ( ) 4 ( ) More than 4

1.11. Number of conforts in your home (beyond the kitchen and bathrooms):

( ) 1 ( ) 2 ( ) 3 ( ) 4 ( ) More than 4

1.12. Residence location :State___________________ Municipal___________________

1.13. History of cancer in the family: are there cases of cancer in your family? ( ) Yes ( ) No

1.14. History of diseases: Were you a healthy person before the diagnosis? ( ) Yes ( ) No

1.15. Do you have constant health problems? ( ) Yes ( ) No

1.16. Did you take any prolonged health treatment? ( ) Yes ( ) No

1.17. When did you received your cancer diagnosis (date)?___________________________________

1.18. Which type of health unit did you receive your cancer diagnosis?

( ) Basic public health unit ( ) Public hospital

( ) Private doctor´s office ( ) Private hospital

1.19. Location of health unit: State __________________ Municipal_______________

1.20. How long was between the diagnosis and treatment beginning? ____________________

1.21. Start of treatmanent (first therapeutic intevention): ____/____/____

1.22. How long was treatment/how much time does treatment?______________________

1.23. Do you continue with the treatment? ( ) Yes ( ) No

1.24. What is your treatment?

( ) Chemotherapy ( ) Radiotherapy ( ) Surgery ( ) Hormiotherapy

1.25. How often did you go to the unit of healty at the treatment phase?_________________________

1.26. Did it brought complications for you? ( ) Yes ( ) No Type:­­­­­­­­­­­­____________________________

1.27. What kind of support was received during your visits to the health unit?

| unity health | ( ) Physical | ( ) Emotional | ( ) social |
| --- | --- | --- | --- |
| family | ( ) Physical | ( ) Emotional | ( ) social |
| civel society | ( ) Physical | ( ) Emotional | ( ) social |
| social protection | ( ) Physical | ( ) Emotional | ( ) social |

1.28. Have your family been informing also about your treatment? ( ) Yes ( ) No

2. Diagnosis of illness (audiography)

2.1. Could you describe for me how you were feeling after the diagnosis (description of physical feelings, emotional and possible changes in time. Did you receive some physical, emotional, financial support (how do you evaluate the weight of each)?

3 . Treatment (audiography)

3.1 do you consider that the treatment was satisfactory? ____________________Why?. (explain the reasons why you think that the treatment was satisfactory or unsatisfactory)?

3.2. Could you tell us about the consequences (positive and negative) that the treatment had in the evolution of your illness and their respective stages?

3.3. For cases of breast cancer: have you been given the opportunity to make a reconstructive surgery? comment your decision.

4. Short and long term experience with cancer (audiography)

4.1. Could you appoint what were the most important (significant) moments for you and your relative during the cancer experience?

4.2. What are the moments that you most remember (explore the meanings)

4.3. Have you stopped smoking or intake alcoholic drink? Have you changed your food habits?

5. Activities before and after treatment (audiography)

5.1. Did you receive any information in the health unit about the changes that your relative would have after (or also during) the treatment? Did you receive any orientation how to deal with these changes?

5.2. Can you tell us how these changes affect your everyday life?

5.3. Do you do any activity that you did not before your cancer diagnosis?

5.4. Can you describe one day of the week and one normal sunday before and after your cancer diagnosis? What has changed?

5.5. Did you return to do all the activities that you did before treatment (individual, family, and social)?

5.6. If you returned to work and / or to go to school, please talk about the possible adaptations that was necessary for that happening (whole or partial time, with mobility and accessibility mechanisms, etc).

5.7Can you tell us about the reaction of your friends in the work and/or school abour your illness?(support, solidarity, etc indifference)?

5.8. did you needed to do any adaptation in your home since the treatment for your better confort and movement (furniture, bathroom, kitchen etc)?

5.9. Did you need to redistribute any of your home tasks between family members (cleaning, shopping, taking children to school etc)?

6. Supports received (audiography)

6.1. Between diagnosis and treatment, what kind of support did you received (of physicians, health units, your family or non-governmental organizations)?

6.2. Who in your family is tasked especially from your care? how?

6.3. Have your spouse participated in your care since the diagnosis?

6.4. How has it been the relationship with his / her spouse after the disease?

6.5. How it been your relationship with other members of your family?

6.6. How do you feel~~s~~ now? How have you been feeling lately? (low or strong, sad or glad?)

6.7. Did they Prescribed to you medications in the health unit? What did they prescribe? Do you take another type of medication?

6.8. Do you receive drugs to feel better? Other treatments? Do you think that you need or need more help from physicians, health units, your family or non-governmental organizations to get better your life today? What kind of help?

7. Expenses with disease (audiography)

7.1. From the diagnosis, have you need to pay for medicines, laboratory, professional support or others?

7.2. How did you make to cover the expenses?

7.3. Does the expenses with disease have affected the economic situation of your family (describe)?

8 – Experience balance: (audiography)

8.1 – Would you know to tell me why you had this disease?

8.2 - do you consider that you had any gain (wide amount)?

8.3 - Would you like to suggest or make any recommendation for the people who treated you and head units and health policies?

8.4 - What would you like to tell to a person who receives today the diagnosis of the disease that you had?
